# Circulating Interleukin-6 Level, Dietary Antioxidant Capacity, and Risk of Colorectal Cancer

**DOI:** 10.3390/antiox8120595

**Published:** 2019-11-28

**Authors:** Jimi Kim, Jeonghee Lee, Jae Hwan Oh, Hee Jin Chang, Dae Kyung Sohn, Aesun Shin, Jeongseon Kim

**Affiliations:** 1 Department of Cancer Biomedical Science, Graduate School of Cancer Science and Policy, National Cancer Center, Goyang-si, Gyeonggi-do 10408, Korea; jmkim@ncc.re.kr (J.K.); jeonghee@ncc.re.kr (J.L.); 2 Center for Colorectal Cancer, National Cancer Center Hospital, National Cancer Center, Goyang-si, Gyeonggi-do 10408, Korea; jayoh@ncc.re.kr (J.H.O.); heejincmd@ncc.re.kr (H.J.C.); gsgsbal@ncc.re.kr (D.K.S.); 3 Department of Preventive Medicine, Seoul National University College of Medicine, 103 Daehak-ro, Jongno-gu, Seoul 03080, Korea; shinaesun@snu.ac.kr; 4 Cancer Research Institute, Seoul National University, 103 Daehak-ro, Jongno-gu, Seoul 03080, Korea

**Keywords:** colorectal cancer, interleukin-6, oxygen radical absorbance capacity, inflammation, antioxidants

## Abstract

Chronic inflammation is one of the causes of colorectal cancer (CRC), and circulating levels of inflammatory biomarkers have been linked to tumor promotion and progression. We aimed to evaluate the interleukin-6 (IL-6) level in CRC patients and determine whether a diet rich in antioxidants was associated with CRC. This study included 654 cases and 1312 controls matched for age and sex. We measured the plasma IL-6 concentration and estimated dietary antioxidant capacity based on oxygen radical absorbance capacity (ORAC) combined with a 106-item semiquantitative food frequency questionnaire. The IL-6 concentration was significantly increased in individuals with CRC (OR _Q4 vs. Q1_, 95% CI = 6.23, 4.10–9.45, *p* < 0.001). High dietary ORAC showed an inverse association with CRC (total ORAC OR _Q4 vs. Q1_, 95% CI = 0.26, 0.16–0.40, *p* < 0.001; total phenolics = 0.32, 0.21–0.50, *p* < 0.001). We found that low dietary ORAC was associated with a significant increase in CRC in the group with elevated IL-6 levels (total ORAC OR _Q4 vs. Q1_, 95% CI = 4.34, 3.12–6.02, *p* < 0.001; total phenolics = 4.61, 3.33–6.39, *p* < 0.001). This study suggested an inverse association between dietary antioxidant capacity and IL-6 level among patients with CRC.

## 1. Introduction

Globally, colorectal cancer (CRC) is the third most common cancer and the second most common cause of death worldwide [1]. It is estimated that approximately 1.8 million new cases of CRC occurred and 881000 people worldwide died from CRC in 2018 [1]. The incidence and mortality patterns and trends in CRC burden have demonstrated that the development of CRC is linked to dietary pattern, lifestyle factors, obesity, family history of CRC, and inflammatory bowel disease (IBD) [1,2]. Moreover, the World Cancer Research Fund/American Institute for Cancer Research (WCRF/AICR) has indicated the known major risk factors for CRC and has revealed that diet, nutrition, and physical activity reduce the risk of CRC [3].

The association of chronic inflammation with CRC has been described in the growing literature. Chronic inflammation may give rise to oncogenesis in the context of CRC via continual unregulated proliferation, apoptosis and genetic mutations [4,5]. The activation of the immune response in immune cells produces inflammatory cytokines that may lead to the development and progression of CRC [6,7]. Chronic inflammation mediated by reactive oxygen or nitrogen species has been linked to the development of sporadic and colitis-associated CRC, leading to the release of several proinflammatory cytokines associated with tumor promotion and progression [8]. Among proinflammatory cytokines, several studies have reported that the pleiotropic cytokine interleukin-6 (IL-6), which is produced by various cell types, contributes to the inhibition of the apoptosis and proliferation of tumor cells [9,10,11]. Many studies have shown a correlation between IL-6 expression and the risk of CRC [5,11]. Increased expression of IL-6 was found both in patients with CRC and in tumor tissue [12,13,14]. Growing evidence suggests that the biological linkage between IL-6 induced signaling and chronic inflammation through oxidative stress resulting from the effect of reactive oxygen species on DNA repair mechanisms resulted in the development of CRC [5,15].

Previous studies have reported that diets rich in antioxidants from fruits and vegetables have shown an inverse association with the risk of CRC [16,17,18,19]. Antioxidants play a role in the prevention of free radical-induced oxidative damage to cells, which means that they neutralize reactive oxygen species [20,21]. An assay of oxygen radical absorbance capacity (ORAC) has been proposed to provide a parameter reflecting antioxidant activities in foods, which describes the cumulative capacity of food components to scavenge free radicals, as well as other synergistic antioxidant effects related inflammatory complications [22,23,24,25]. Several epidemiological studies have shown the associations between total antioxidant capacity based on ORAC and health benefits, although the results were inconsistent depending on the type of disease [26,27,28]. However, no study has yet assessed the associations between dietary ORAC and CRC risk underlying the plasma IL-6 level, which is regarded as a tumor-promoting biomarker.

Therefore, the objectives of this study were, first, to estimate the plasma IL-6 level associated with CRC risk; second, to compare the dietary ORAC between controls and CRC cases; and third, to identify the associations between dietary ORAC, the plasma IL-6 levels, and the risk of CRC in a Korean population.

## 2. Materials and Methods

### 2.1. Study Population

In this study, participants were recruited from the Center for Colorectal Cancer of the National Cancer Center in Korea in cooperation with the Center for Cancer Prevention and Detection at the same hospital; participants attended the center either for a health check-up or for a CRC diagnosis. Patients who were newly diagnosed with CRC by pathological confirmation of adenocarcinoma via endoscopic biopsies were recruited from August 2010 to August 2013. We used the Korea Central Cancer Registry (KCCR) database to determine whether participants had cancer and then selected cancer-free controls between October 2007 and December 2014. All of the participants were asked to provide a questionnaire on demographics and lifestyles, a 106-item semiquantitative food frequency questionnaire (SQFFQ), and a blood sample. Of the participants in this study, including 1070 cases and 14201 controls, those with an incomplete demographic questionnaire or SQFFQ and those reporting implausible energy intake (<500 kcal/day or >4000 kcal/day) were excluded. Among the participants enrolled in this study, we analyzed-plasma IL-6 biomarker in 2531 subjects including 696 cases and 1835 controls. We used the frequency matching method between the cases and controls at a ratio of 1:2 by 5-year age group and sex. Consequently, the total number of participants was 1966 including 654 cases and 1312 controls after removing outliers (<0.5%, >99.5%) of plasma levels of IL-6 biomarker (Figure S1). We used the International Statistical Classification of Disease and Related Health Problems 10th revision (ICD-10) to classify the anatomical location of CRC. The anatomic sites were categorized into two groups as colon cancer combined with proximal colon and distal colon and rectal cancer: Proximal colon (cecum, ascending colon, hepatic flexure, transverse colon, and splenic flexure); distal colon (descending colon, sigmoid-descending colon junction, and sigmoid colon); and rectum (rectosigmoid colon and rectum). The study was approved by the Institutional Review Board of the National Cancer Center Korea (IRB No.NCCNCS-10-350 and No.NCC2015-0202). All participants signed an informed consent form before participation.

### 2.2. Dietary Assessment and Dietary Antioxidant Capacity Based on the ORAC Database

Dietary intake data of participants were obtained using a validated 106-item SQFFQ from a well-trained interviewer [29]. The SQFFQ included the portion size (small, medium, large) and frequency of consumption (never or rarely, 1 time per month, 2–3 times per month, 1–2 times per week, 3–4 times per week, 5–6 times per week, 1 time per day, 2 times per day, 3 times per day) for each food item consumed within the past 12 months. Analysis of the SQFFQ including the nutrient and energy content of each food item was performed using CAN-PRO 4.0 (Computer Aided Nutritional analysis program, Korean Nutrition Society, Seoul, Korea).

To calculate the dietary total antioxidant capacity for each participant, we estimated the levels of hydrophilic ORAC (H-ORAC), lipophilic ORAC (L-ORAC), total ORAC (T-ORAC), and total phenolics (TPs) from the United States Department of Agriculture (USDA) Database for *Oxygen Radical Absorbance Capacity (ORAC) of Selected Foods, Release 2* (www.ars.usda.gov) [25]. The individual indices of ORAC were reported in μmol of Trolox equivalents per 100 g (μmol TE/100 g) for H-ORAC, L-ORAC, and T-ORAC and in mg gallic acid equivalents per 100 g (mgGAE/100 g) for TPs. In terms of matching between a 106-item of the SQFFQ and ORAC database, 56 food items from the SQFFQ had antioxidant contents, and these items consisted mainly of fruits, vegetables, nuts, legumes, and tea. The validity and reproducibility of the findings showed that plasma ORAC was correlated with FFQ-based ORAC intake, providing FFQ-based ORAC values to assess antioxidant intake from foods [30].

### 2.3. Laboratory Assays for IL-6 Biomarker Concentration

The plasma from the blood specimens obtained from each participant was separated and stored at −80 ℃. Plasma concentrations of IL-6 (pg/mL) were measured using a commercially available Quantikine HS human IL-6 Immunoassay with Enzyme-Linked Immunosorbent Assay (ELISA) according to the manufacturer’s recommendations (R&D Systems, Minneapolis, MN, USA).

### 2.4. Statistical Analysis

The *t*-test and the *x*^2^ test were used for the comparison of general characteristics between cases and controls, and we use mean and standard deviation (SD) or percentage and number to describe the results. The distribution of plasma IL-6 levels and dietary ORAC are presented as the median and interquartile range (IQR) and were assessed by using the Wilcoxon signed-rank test. The values of IL-6 were log-transformed to improve the normality of the distribution. We used the receiver operating characteristics (ROC) curve for IL-6 to determine the diagnostic ability between the two groups. We conducted the analysis of contributing foods of each dietary ORAC represented up to 90% of the cumulative contribution among 56 food items. Energy adjustment of dietary ORAC, including H-ORAC, L-ORAC, T-ORAC, TPs, and their contributing foods, was performed using the residual method [31]. We analyzed odds ratios (ORs) and 95% confidence intervals (CIs) using unconditional logistic regression models and expressed ORs per quartile of IL-6 concentration and dietary ORAC based upon the quartile distribution among controls. To analyze the associations between dietary ORAC and the levels of IL-6, we divided each subtype of ORAC (low/high) into two groups based on the median levels of the control. The multivariable models were adjusted for age, sex, BMI, education level, occupation, income, smoking status, alcohol consumption status, physical activity, first-degree family history of CRC, and total energy intake. We used a multinomial logistic regression to compare controls and individuals with each anatomic site subtype of CRC. All statistical tests were two-sided, and all analyses were performed using SAS version 9.4 software (SAS Institute Inc., Cary, NC, USA).

## 3. Results

The sociodemographic and lifestyle characteristics of the participants are shown in Table 1. When comparing two groups, the control group demonstrated a higher education level, prevalence of a professional occupation, income, and prevalence of regular exercise and a lower percentage of ex-alcohol consumers and individuals with a first-degree family history of CRC than the case group (*p* < 0.05). The controls had a significantly lower intake of total energy than the cases (1693.04 ± 562.86 vs. 2022.17 ± 525.74, *p* < 0.001). With respect to the dietary ORAC from the SQFFQ, we observed that the mean values of H-ORAC (μmolTE/d), L-ORAC (μmolTE/d), T-ORAC (μmolTE/d), and TPs (mgGAE/d) were significantly lower in cases (H-ORAC: 4870.43 ± 3806.52 vs. 3323.50 ± 2008.79, L-ORAC: 196.45 ± 116.62 vs. 147.06 ± 75.94, T-ORAC: 5062.87 ± 3863.00 vs. 3467.73 ± 2048.26, TPs: 452.36 ± 386.38 vs. 301.44 ± 222.92, *p* < 0.001). For the biomarker analysis, the plasma concentration of IL-6 (pg/mL) was significantly higher in the cases (3.64 ± 5.68) than in the controls (1.93 ± 2.39) (*p* < 0.001). The median levels of IL-6 was significantly different between the cases and the controls (1.36 vs. 2.14, *p* < 0.001). We assessed the correlation between dietary ORAC and IL-6 biomarker in all study participants at baseline (Table S1). There were inverse correlations of H-ORAC (*r* = −0.116, *p* < 0.001), L-ORAC (*r* = −0.097, *p* < 0.001), T-ORAC (*r* = −0.116, *p* < 0.001), and TPs (*r* = −0.123, *p* < 0.001) with plasma IL-6 concentration. Additionally, to determine the optimal cut-off values for CRC between the two diagnostic groups, we calculated the area under the ROC curve (AUC) with the sensitivity and specificity of the CRC diagnosis as a biomarker (Figure S2). The AUC for the plasma IL-6 concentration was 0.70 and a cut-off value (sensitivity, 1- specificity) was 1.62 (0.68, 0.63) indicating a moderation of CRC diagnostic accuracy.

Table 2 shows the associations between quartiles of the plasma IL-6 concentration and the risk of CRC. A significant association was observed between the IL-6 levels and the risk of CRC in the highest quartile of IL-6 compared to the lowest quartile after adjusting for the confounding variables (OR _Q4 vs. Q1_, 95% CI = 6.23, 4.10–9.45, *p* < 0.001). When stratified by anatomic site of CRC, we observed a significantly increased the risk of colon cancer (OR _Q4 vs. Q1_, 95% CI = 4.77, 2.93–7.76, *p* < 0.001), as well as rectal cancer (OR _Q4 vs. Q1_, 95% CI = 8.06, 4.52–14.39, *p* < 0.001), after considering the aforementioned confounding variables.

Table 3 shows the associations between dietary ORAC and the risk of CRC. A significantly decreased the risk of CRC was observed in individuals with higher dietary ORAC and its subtypes in multivariable models as follows: H-ORAC (OR _Q4 vs. Q1_, 95% CI = 0.29, 0.19–0.44, *p* < 0.001); L-ORAC (OR _Q4 vs. Q1_, 95% CI = 0.18, 0.12–0.29, *p* < 0.001); T-ORAC (OR _Q4 vs. Q1_, 95% CI = 0.26, 0.16–0.40, *p* < 0.001); and TPs (OR _Q4 vs. Q1_, 95% CI = 0.32, 0.21–0.50, *p* < 0.001). When the results were stratified by anatomic site, we observed a significantly decreased the risk of colon cancer associated with dietary ORAC in the multivariable models as follows: H-ORAC (OR _Q4 vs. Q1_, 95% CI = 0.30, 0.18–0.51, *p* < 0.001); L-ORAC (OR _Q4 vs. Q1_, 95% CI = 0.15, 0.08–0.27, *p* < 0.001); T-ORAC (OR _Q4 vs. Q1_, 95% CI = 0.25, 0.15–0.44, *p* < 0.001); and TPs (OR _Q4 vs. Q1_, 95% CI = 0.35, 0.21–0.61, *p* < 0.001). In addition, we found similar associations of dietary ORAC with the risk of rectal cancer in the multivariable models as follows: H-ORAC (OR _Q4 vs. Q1_, 95% CI = 0.27, 0.15–0.47, *p* < 0.001); L-ORAC (OR _Q4 vs. Q1_, 95% CI = 0.23, 0.13–0.40, *p* < 0.001); T-ORAC (OR _Q4 vs. Q1_, 95% CI = 0.24, 0.14–0.43, *p* < 0.001); and TPs (OR _Q4 vs. Q1_, 95% CI = 0.29, 0.16–0.52, *p* < 0.001).

Table 4 shows the associations between dietary ORAC and plasma levels of IL-6 in terms of the risk of CRC. According to the subgroups of dietary ORAC, we found that a high level of IL-6 was strongly associated with an increased the risk of CRC in the lower intake of H-ORAC, L-ORAC, T-ORAC, and TPs groups in the comparison between the highest and lowest quartiles as follows: H-ORAC (OR _Q4 vs. Q1_, 95% CI = 4.26, 3.07–5.91, *p* < 0.001); L-ORAC (OR _Q4 vs. Q1_, 95% CI = 4.70, 3.38–6.52, *p* < 0.001); T-ORAC (OR _Q4 vs. Q1_, 95% CI = 4.34, 3.12–6.02, *p* < 0.001); and TPs (OR _Q4 vs. Q1_, 95% CI = 4.61, 3.33–6.39, *p* < 0.001), after adjusting for the aforementioned confounding variables.

## 4. Discussion

In the present study, we demonstrated that CRC occurrence increased as the concentration of plasma IL-6 increased, and high dietary ORAC was associated with a decreased the risk of CRC in a Korean population. Moreover, we observed inverse associations between dietary antioxidant capacity and plasma IL-6 level among patients with CRC.

Concerning CRC, chronic inflammation is an important risk factor for tumor development [32]. Active inflammation in colorectal mucosa results in the development of CRC through long-term colitis that may affect immune cells and their products [4,32]. Many of the molecular alterations are linked to the inflammatory process involved in CRC carcinogenesis; these alterations involve cyclooxygenase-2 (COX-2) and nuclear factor kappa B (NF-kB) genes, which are related to the regulation of the expression of various cytokines and oxidative stress that contribute to neoplastic transformation [4,5]. Growing evidence has reported that the cytokines released by activated tumor cells play a role in tumor progression by promoting the growth, differentiation, and survival of tumor cells [33]. Moreover, in terms of cytokines, an inflamed epithelium produces excessive reactive oxygen and nitrogen species that affect colorectal tumorigenesis including tumor promotion and progression by genetic alteration [33,34]. As a proinflammatory cytokine, IL-6 and IL-6 signaling are associated with CRC development [11,35]. Several studies have reported that higher circulating IL-6 levels were significantly correlated with CRC status, demonstrating associations with tumor size, stage, and metastasis [12,13,36]. In addition, a recent systematic review described that the IL-6 levels were linked to short overall survival including in patients with CRC, suggesting its use as a prognostic biomarker for CRC [37]. In this study, we analyzed the AUC, sensitivity and specificity between plasma levels of IL-6 and CRC, providing evidence for its use as a biomarker for CRC diagnosis. Furthermore, we also observed that plasma IL-6 levels were significantly higher in patients with CRC than in controls, revealing associations between IL-6 levels and the risk of CRC. Accumulating evidence has demonstrated that IL-6 produced by inflamed epithelial cells mediates the promotion of tumorigenesis by activating the downstream Janus kinases (JAKs) and signal transducer and activator of transcription 3 (STAT3) [10,11,12]. 

In the tumor microenvironment, the activated inflammatory cells produce not only inflammatory cytokines but also reactive oxygen and nitrogen species, leading to DNA damage and mutation [34]. The inflammatory cytokines may stimulate the generation of reactive oxygen species in inflamed cells, resulting in epigenetic alterations, genetic instability, aberrant methylation, and carcinogenesis [5,34]. Several studies have demonstrated that the reactive oxygen species released by epithelia and immune cells may have mechanistic links including COX-2, NF-kB, p53, DNA mismatch repair genes, and others [5].

To prevent the development of carcinogenesis by free radicals and reactive oxygen species, a wide range of antioxidants and antitumor activities are required [34,38]. Numerous studies have been performed on the effects of antioxidants derived from various types of food in regard to their antitumor properties [39]. The ORAC database is one of the USDA National Nutrient Databases focused on the degree of inhibition of peroxy-radical-induced oxidation associated with foods [25]. Several studies have shown that ORAC from foods may have associations with preventing the development of human diseases. Dietary ORAC showed an inverse association with hypertension in type 2 diabetic patients in a cross-sectional study, suggesting that dietary antioxidants may decrease the risk of hypertension [26]. In particular, phenolic intake, based on the ORAC database, decreased the risk of endometrial cancer, revealing the role of antioxidants in preventing carcinogenesis [27]. However, the intake of antioxidants, based on ORAC, was not associated with the risk of preeclampsia, although the serum total antioxidant capacity showed a positive association with preeclampsia [28]. In this study, we used a 106-item SQFFQ with the ORAC database and then analyzed the antioxidant activities of fruits, vegetables, nuts, legumes, and tea. We observed a decreased the risk of CRC when comparing the highest quartile of dietary ORAC with the lowest quartile.

Antioxidants are abundant in not only fruits, vegetables, legumes, nuts, and wine but also tea, providing evidence that a high content of antioxidants derived from foods may have an impact on the prevention of cancer, including CRC [39]. Regarding CRC caused by oxidative stress, there are numerous studies showing that the consumption of foods rich in antioxidants can affect the risk of CRC [40]. Some of the most common antioxidants are polyphenols, tocopherols, carotenoids, curcumin, and vitamin C, which provide antioxidant effects, including the regulation of cellular proliferation, apoptosis, and gene expression in the context of colorectal carcinogenesis [40,41]. In this study, when we compared the major consumption of foods contributing to H-ORAC, L-ORAC, T-ORAC, and TPs between the controls and cases, it shows the different major components in accordance with H-ORAC, L-ORAC, T-ORAC, and TPs. In H-ORAC, the cases were more likely to have less intake of green tea, grapes, apples, strawberries, sweet potatoes, radishes, potatoes, plums, bananas, peaches, soybeans, tomato juice, lettuce, and lemon juice than controls (*p* < 0.05) (Table S2). In L-ORAC, the cases consumed less red pepper, bananas, sweet potatoes, black pepper, potatoes, lettuce, apples, peanuts, garlic, radishes, mushroom, watermelon, strawberries, peaches and carrots than controls (*p* < 0.05) (Table S3). The results of T-ORAC showed the similar major components with H-ORAC and L-ORAC (Table S4). In TPs, the cases had a lower intake of tomato juice, apples, potatoes, bananas, strawberries, sweet potatoes, cabbages, radishes, catsup, watermelon, raisins, plums, peanuts, peaches, lemon juice, lettuce, red pepper, and soybeans than controls (*p* < 0.05) (Table S5). Although some epidemiological studies have reported conflicting data, there is strong mechanistic evidence for the effect of antioxidants on CRC induced by reactive oxygen species, demonstrating protective mechanisms that neutralize the production of free radicals [42,43,44].

Although we controlled for potential confounding factors including family history of CRC and modifiable lifestyle factors in this study, it should be mentioned regarding other factors affecting the inflammation of gastrointestinal (GI) tract and cancer. During decades, many evidences have determined that inflammation plays a role in the development of cancer and is involved in all stages of tumorigenesis [45,46]. There are several stimuli of inflammation of GI tract including somatic mutations and environmental factors such as dietary factors, obesity, tobacco smoking, pollutants, and medications resulted in DNA damage, genomic instability, and tumor promotion [46]. As the result of inflammation, different types of immune cells are in the process of either tumor promoting mechanisms or anti-tumorigenic mechanisms by producing the reactive oxygen species, reactive nitrogen intermediates, cytokines, and chemokines in tumor initiation and promotion [46,47]. Among the dietary factors, numerous studies have reported that phytochemicals such as flavonoids rich in fruits and vegetables mainly contributed to regulation of immune system via production of antioxidants and scavenging activity [48,49,50]. Previous epidemiological studies have reported the associations between dietary antioxidants and cancer suggesting the role of the inflammation [50,51]. In this study, we examined the dietary antioxidant capacity and plasma IL-6 level, one of the major tumor-promoting cytokines, regarding risk of CRC. Given that inflammation involves complex cross-talk and interaction into molecular and cellular mechanisms with all stages of tumor development, future studies with a prospective design are needed to achieve the understanding of associations between dietary antioxidant capacity, IL-6 level, and risk of CRC with comprehensive factors in the inflammatory tumor microenvironment.

This study has several strengths. We used a comprehensive and validated SQFFQ and then calculated dietary ORAC including the contents of fruits, vegetables, nuts, legumes, and tea. To the best of our knowledge, no study has yet reported the associations between dietary ORAC and the risk of CRC. This study attempted to use a parameter reflecting antioxidant activities in diet, providing comparison of food components to scavenge free radicals and other synergistic antioxidant effects. We examined whether the elevated level of plasma IL-6 was associated with an increased the risk of CRC and assessed its role as a proinflammatory biomarker in CRC carcinogenesis. Therefore, this study determined not only the association between diets rich in antioxidant activities and CRC risk, but also the measurement of plasma IL-6 level as a possible correlation between them. This study suggests that further study is necessary to explore the molecular and cellular mechanism based on the associations between dietary antioxidant capacity, IL-6 level, and risk of CRC.

There are limitations to this study. Because of the hospital-based case-control design, potential selection bias and recall bias should be considered. The control groups recruited from a health check-program underwent a health screening and therefore may have had healthier lifestyle and dietary habits than patients with CRC. In addition, we matched a 106-item SQFFQ with the USDA ORAC database, which included several contends, so dietary ORAC might be insufficiently covered for all items reported in the SQFFQ. Moreover, the sample size was relatively small to explore the interactive associations between dietary antioxidant capacity, IL-6 level, and the risk of CRC.

## 5. Conclusions

In conclusion, higher dietary ORAC was inversely associated with the risk of CRC in the subgroup with elevated plasma IL-6 levels. Given that there were associations between plasma IL-6 concentration and CRC risk, we suggest that plasma IL-6 level is a possible intermediate biomarker for diagnosis of CRC. This study provides the further evidence regarding the associations between dietary antioxidant capacity, IL-6 level, and risk of CRC. In addition, this study may provide the importance of consideration on antioxidant capacity derived from foods to establish the more effective dietary guidelines and public health policy to reduce the risk of CRC.

## Figures and Tables

**Table 1:** General characteristics of study participants.

	Controls (*n* = 1312)	Cases (*n* = 654)	*p*-Value ^a^
Age (years)			
Mean ± SD	56.10 ± 9.21	56.44 ± 9.60	0.45
Sex (*n*, %)			
Male	895 (68.22)	446 (68.20)	>0.99
Female	417 (31.78)	208 (31.80)	
Body mass index (BMI, kg/m^2^) (*n*, %)			
Mean ± SD	24.26 ± 2.88	24.14 ± 3.52	0.44
<25	828 (63.03)	411 (62.84)	0.92
≥25	484 (36.89)	243 (37.16)	
Education level (*n*, %)			
Middle school or less	201 (15.32)	241 (36.85)	<0.001
High school	422 (32.16)	248 (37.92)	
College or more	658 (50.15)	165 (25.23)	
Occupation (*n*, %)			
Professionals, administrative, management, office jobs	339 (25.84)	136 (20.80)	<0.001
Sales and service positions	284 (21.65)	27 (4.13)	
Agriculture, manufacturing, mining, army service	165 (12.58)	108 (16.51)	
Housekeeping, unemployment, and others	510 (38.87)	383 (58.56)	
Income (10,000 won/month) (*n*, %)			
<200	284 (21.65)	226 (34.56)	<0.001
200–400	536 (40.85)	428 (65.44)	
>400	379 (28.89)	0 (0.00)	
Smoking status (*n*, %)			
None	575 (43.83)	298 (45.57)	0.05
Ex-smoker	496 (37.80)	214 (32.72)	
Current-smoker	241 (18.37)	142 (21.71)	
Alcohol drinking status (*n*, %)			
None	406 (30.95)	200 (30.58)	0.025
Ex-drinker	121 (9.22)	86 (13.15)	
Current-drinker	785 (59.83)	368 (56.27)	
Physical activity status (*n*, %)			
Yes	759 (57.85)	207 (31.65)	<0.001
No	545 (41.54)	447 (68.35)	
First-degree family history of colorectal cancer (CRC) (*n*, %)			
Yes	73 (5.56)	57 (8.72)	0.008
No	1239 (94.44)	597 (91.28)	
Total caloric intake (kcal/day)			
Mean ± SD	1693.04 ± 562.86	2022.17 ± 525.74	<0.001
semiquantitative food frequency questionnaire (SQFFQ)- oxygen radical absorbance capacity (ORAC)			
hydrophilic ORAC (H-ORAC) (μmolTE/d)			
Mean ± SD ^b^	4870.43 ± 3806.52	3323.50 ± 2008.79	<0.001 ^d^
Median	3796.64	2837.11	
(interquartile range (IQR)) ^c^	(2350.58, 6181.32)	(1961.12, 4185.10)	
lipophilic ORAC (L-ORAC) (μmolTE/d)			
Mean ± SD ^b^	196.45 ± 116.62	147.06 ± 75.94	<0.001 ^d^
Median	173.96	134.98	
(IQR) ^c^	(120.81, 248.60)	(96.27, 183.24)	
total ORAC (T-ORAC) (μmolTE/d)			
Mean ± SD ^b^	5062.87 ± 3863.00	3467.73 ± 2048.26	<0.001 ^d^
Median	4022.77	2987.32	
(IQR) ^c^	(2485.96, 6465.70)	(2080.73, 4363.09)	
TPs (mgGAE/d)			
Mean ± SD ^b^	452.36 ± 386.38	301.44 ± 222.92	<0.001 ^d^
Median	344.79	242.71	
(IQR) ^c^	(195.37, 581.23)	(168.91, 363.96)	
IL-6 (pg/mL)			
Mean ± SD	1.93 ± 2.39	3.64 ± 5.68	<0.001 ^d^
Median	1.36	2.14	
(IQR) ^c^	(0.98, 2.04)	(1.42, 3.46)	

ORAC: Oxygen radical absorbance capacity; H-ORAC: Hydrophilic oxygen radical absorbance capacity; L-ORAC: Lipophilic oxygen radical absorbance capacity; T-ORAC: Total oxygen radical absorbance capacity; TPs: Total phenolics; TE: Trolox equivalents; GAE: Gallic acid equivalents. ^a^ *P*-values were calculated using the χ^2^ test for categorical variables and the *t*-test for continuous variables. ^b^ Dietary ORAC was adjusted for total energy intake using the residual method. ^c^ IQR, interquartile ranges. ^d^ Wilcoxon test was used for significant *p*-values that met the 5% level.

**Table 2:** Association between plasma IL-6 concentration and CRC risk stratified by anatomic site.

	Plasma IL-6 Concentration (pg/mL)				
	Q1 (<0.98)	Q2 (0.98 to <1.36)	Q3 (1.36 to <2.04)	Q4 (≥2.04)	P for Trend
Colorectal cancer					
No. controls/cases	328/52	328/94	328/154	328/354	
Crude OR (95% CI)	1.0 (ref)	1.81 (1.25–2.62)	2.96 (2.09–4.20)	6.81 (4.90–9.46)	<0.001
Multivariable OR (95% CI) ^a^	1.0 (ref)	1.97 (1.24–3.11)	2.82 (1.82–4.37)	6.23 (4.10–9.45)	<0.001
Colon cancer					
No. controls/cases	328/32	328/46	328/80	328/176	
Crude OR (95% CI)	1.0 (ref)	1.44 (0.89–2.31)	2.50 (1.61–3.87)	5.50 (3.66–8.26)	<0.001
Multivariable OR (95% CI) ^a^	1.0 (ref)	1.63 (0.94–2.84)	2.33 (1.39–3.92)	4.77 (2.93–7.76)	<0.001
Rectal cancer					
No. controls/cases	328/20	328/48	328/72	328/168	
Crude OR (95% CI)	1.0 (ref)	2.40 (1.39–4.13)	3.60 (2.14–6.05)	8.40 (5.16–13.69)	<0.001
Multivariable OR (95% CI) ^a^	1.0 (ref)	2.42 (1.29–4.55)	3.48 (1.90–6.39)	8.06 (4.52–14.39)	<0.001

Q, quartile. REF: Reference. ^a^ Multivariable odds ratio (OR) was adjusted for age, sex, BMI, education level, occupation, income, smoking status, alcohol consumption, physical activity, first-degree family history of CRC, and total energy intake. Significant *p*-values that met the 5% level.

**Table 3:** Association between dietary ORAC and CRC risk stratified by anatomic site.

			Dietary ORAC		
	Q1	Q2	Q3	Q4	*P* for Trend
Colorectal Cancer					
H-ORAC (μmolTE/d)	<2350.58	2350.58 to <3796.64	3796.64 to <6181.33	≥6181.32	
No. controls/cases	328/242	328/211	328/147	328/54	
Crude OR (95% CI)	1.0 (ref)	0.87 (0.69–1.11)	0.61 (0.47–0.78)	0.22 (0.16–0.31)	<0.001
Multivariable OR (95% CI) ^a^	1.0 (ref)	0.94 (0.69–1.29)	0.68 (0.48–0.96)	0.29 (0.19–0.44)	<0.001
L-ORAC (μmolTE/d)	<120.81	120.81 to <173.96	173.96 to <248.60	≥248.60	
No. controls/cases	328/277	328/193	328/141	328/43	
Crude OR (95% CI)	1.0 (ref)	0.70 (0.55–0.89)	0.51 (0.40–0.66)	0.16 (0.11–0.22)	<0.001
Multivariable OR (95% CI) ^a^	1.0 (ref)	0.82 (0.60–1.13)	0.59 (0.42–0.83)	0.18 (0.12–0.29)	<0.001
T-ORAC (μmolTE/d)	2485.96	2485.96 to <4022.77	4022.77 to <6465.70	≥6465.70	
No. controls/cases	328/248	328/207	328/148	328/51	
Crude OR (95% CI)	1.0 (ref)	0.84 (0.66–1.06)	0.60 (0.46–0.77)	0.21 (0.15–0.29)	<0.001
Multivariable OR (95% CI) ^a^	1.0 (ref)	0.92 (0.67–1.27)	0.68 (0.48–0.96)	0.26 (0.16–0.40)	<0.001
TPs (mgGAE/d)	195.37	195.37 to <344.79	344.79 to <581.24	≥581.24	
No. controls/cases	328/231	328/246	328/122	328/55	
Crude OR (95% CI)	1.0 (ref)	1.07 (0.84–1.35)	0.53 (0.40–0.69)	0.24 (0.17–0.33)	<0.001
Multivariable OR (95% CI) ^a^	1.0 (ref)	1.23 (0.90–1.67)	0.54 (0.38–0.78)	0.32 (0.21–0.50)	<0.001
Colon Cancer					
H-ORAC (μmolTE/d)					
No. controls/cases	328/111	328/118	328/76	328/29	
Crude OR (95% CI)	1.0 (ref)	1.06 (0.79–1.44)	0.69 (0.49–0.95)	0.26 (0.17–0.40)	<0.001
Multivariable OR (95% CI) ^a^	1.0 (ref)	1.19 (0.81–1.73)	0.67 (0.44–1.03)	0.30 (0.18–0.51)	<0.001
L-ORAC (μmolTE/d)					
No. controls/cases	328/131	328/107	328/76	328/20	
Crude OR (95% CI)	1.0 (ref)	0.82 (0.61–1.10)	0.58 (0.42–0.80)	0.15 (0.09–0.25)	<0.001
Multivariable OR (95% CI) ^a^	1.0 (ref)	1.01 (0.70–1.47)	0.62 (0.41–0.93)	0.15 (0.08–0.27)	<0.001
T-ORAC (μmolTE/d)					
No. controls/cases	328/116	328/114	328/77	328/27	
Crude OR (95% CI)	1.0 (ref)	0.98 (0.73–1.33)	0.66 (0.48–0.92)	0.23 (0.15–0.36)	<0.001
Multivariable OR (95% CI) ^a^	1.0 (ref)	1.13 (0.78–1.65)	0.66 (0.43–1.00)	0.25 (0.15–0.44)	<0.001
TPs (mgGAE/d)					
No. controls/cases	328/102	328/144	328/57	328/31	
Crude OR (95% CI)	1.0 (ref)	1.41 (1.05–1.90)	0.56 (0.39–0.80)	0.30 (0.20–0.47)	<0.001
Multivariable OR (95% CI) ^a^	1.0 (ref)	1.65 (1.14–2.40)	0.51 (0.32–0.80)	0.35 (0.21–0.61)	<0.001
Rectal Cancer					
H-ORAC (μmolTE/d)					
No. controls/cases	328/125	328/90	328/70	328/23	
Crude OR (95% CI)	1.0 (ref)	0.72 (0.53–0.98)	0.56 (0.40–0.78)	0.18 (0.12–0.29)	<0.001
Multivariable OR (95% CI) ^a^	1.0 (ref)	0.77 (0.52–1.14)	0.67 (0.43–1.02)	0.27 (0.15–0.47)	<0.001
L-ORAC (μmolTE/d)					
No. controls/cases	328/138	328/84	328/63	328/23	
Crude OR (95% CI)	1.0 (ref)	0.61 (0.45–0.83)	0.46 (0.33–0.64)	0.17 (0.10–0.27)	<0.001
Multivariable OR (95% CI) ^a^	1.0 (ref)	0.73 (0.50–1.08)	0.64 (0.42–0.97)	0.23 (0.13–0.40)	<0.001
T-ORAC (μmolTE/d)					
No. controls/cases	328/126	328/90	328/70	328/22	
Crude OR (95% CI)	1.0 (ref)	0.71 (0.52–0.98)	0.56 (0.40–0.77)	0.18 (0.11–0.28)	<0.001
Multivariable OR (95% CI) ^a^	1.0 (ref)	0.77 (0.52–1.14)	0.69 (0.45–1.06)	0.24 (0.14–0.43)	<0.001
TPs (mgGAE/d)					
No. controls/cases	328/123	328/99	328/63	328/23	
Crude OR (95% CI)	1.0 (ref)	0.81 (0.59–1.09)	0.51 (0.37–0.72)	0.19 (0.12–0.30)	<0.001
Multivariable OR (95% CI) ^a^	1.0 (ref)	0.94 (0.64–1.38)	0.55 (0.36–0.86)	0.29 (0.16–0.52)	<0.001

Q, quartile. ORAC: Oxygen radical absorbance capacity; H-ORAC: Hydrophilic oxygen radical absorbance capacity; L-ORAC: Lipophilic oxygen radical absorbance capacity; T-ORAC: Total oxygen radical absorbance capacity; TPs: Total phenolics; TE: Trolox equivalents; GAE: Gallic acid equivalents; REF: Reference. ^a^ Multivariable odds ratio (OR) was adjusted for age, sex, BMI, education level, occupation, income, smoking status, alcohol consumption, physical activity, first-degree family history of CRC, and total energy intake. Significant *p*-values that met the 5% level.

**Table 4:** Association between dietary ORAC and plasma IL-6 concentration regarding to CRC risk.

	Plasma IL-6 Concentration (pg/mL)							
	Q1 (<0.98)		Q2 (0.98 to <1.36)		Q3 (1.36 to <2.04)		Q4 (≥2.04)	
	No. Controls/Cases	Multivariable OR (95% CI) ^a^	No. Controls/Cases	Multivariable OR (95% CI) ^a^	No. Controls/Cases	Multivariable OR (95% CI) ^a^	No. Controls/Cases	Multivariable OR (95% CI) ^a^
H-ORAC (μmolTE/d)								
Low	157/31	1.0 (ref)	156/67	1.54 (1.02–2.33)	168/102	1.96 (1.35–2.85)	175/253	4.26 (3.07–5.91)
High	171/21	1.0 (ref)	172/27	0.33 (0.20–0.56)	160/52	0.57 (0.36–0.88)	153/101	1.23 (0.86–1.77)
L-ORAC (μmolTE/d)								
Low	148/33	1.0 (ref)	172/69	1.56 (1.04–2.34)	164/115	2.21 (1.53–3.17)	172/253	4.70 (3.38–6.52)
High	180/19	1.0 (ref)	156/25	0.33 (0.19–0.56)	164/39	0.43 (0.27–0.70)	156/101	1.10 (0.77–1.59)
T-ORAC (μmolTE/d)								
Low	155/31	1.0 (ref)	157/66	1.53 (1.01–2.31)	170/105	2.05 (1.41–2.98)	174/253	4.34 (3.12–6.02)
High	173/21	1.0 (ref)	171/28	0.35 (0.21–0.58)	158/49	0.53 (0.34–0.83)	154/101	1.22 (0.85–1.75)
TPs (mgGAE/d)								
Low	142/36	1.0 (ref)	160/73	1.80 (1.20–2.71)	174/102	2.18 (1.49–3.18)	180/266	4.61 (3.33–6.39)
High	186/16	1.0 (ref)	168/21	0.25 (0.14–0.44)	154/52	0.53 (0.34–0.83)	148/88	1.13 (0.77–1.66)

ORAC: Oxygen radical absorbance capacity; H-ORAC: Hydrophilic oxygen radical absorbance capacity; L-ORAC: Lipophilic oxygen radical absorbance capacity; T-ORAC: Total oxygen radical absorbance capacity; TPs: Total phenolics; TE: Trolox equivalents; GAE: Gallic acid equivalents; REF: Reference. Dietary ORAC was categorized into low and high groups based on the median level of their control group’s intake (H-ORAC: 3796.64 μmolTE/d, L-ORAC: 173.96 μmolTE/d, T-ORAC: 4022.77 μmolTE/d, TPs: 344.79 mgGAE/d). ^a^ Multivariable odds ratio (OR) was adjusted for age, sex, BMI, education level, occupation, income, smoking status, alcohol consumption, physical activity, first-degree family history of CRC, and total energy intake. Significant *p*-values that met the 5% level.

## Supplementary Materials

Supplementary data related to this article can be found at online https://www.mdpi.com/2076-3921/8/12/595/s1. Figure S1: Flow chart of study participants, Figure S2: The receiver operating characteristics (ROC) curve for IL-6, Table S1: Spearman correlation coefficient of plasma IL-6 level and dietary ORAC in study participants, Table S2: Comparison of the consumption of dietary H-ORAC contributing foods, Table S3: Comparison of the consumption of dietary L-ORAC contributing foods, Table S4: Comparison of the consumption of dietary T-ORAC contributing foods, Table S5: Comparison of the consumption of dietary TPs contributing foods.
