# Supplementary material for: Circulating Interleukin-6 Level, Dietary Antioxidant Capacity, and Risk of Colorectal Cancer
Source: Antioxidants (Basel). 2019 Nov 28;8(12):595. doi: 10.3390/antiox8120595 (PMC6943549; doi:10.3390/antiox8120595)
Supplement: Supplementary file 1 [file antioxidants-08-00595-s001.zip › 191127_Supplementary Tables_jmk.docx]

**Supplementary Table 1. Spearman correlation coefficient of plasma IL-6 levels and dietary ORAC in study participants**

| **Variable** | **IL-6** | **H-ORAC** | **L-ORAC** | **T-ORAC** | **TPs** |
| --- | --- | --- | --- | --- | --- |
| **IL-6** | 1 | -0.116 ^a^ | -0.097 ^a^ | -0.116 ^a^ | -0.123 ^a^ |
| **H-ORAC** |  | 1 | 0.660 ^a^ | 0.999 ^a^ | 0.912 ^a^ |
| **L-ORAC** |  |  | 1 | 0.681 ^a^ | 0.639 ^a^ |
| **T-ORAC** |  |  |  | 1 | 0.911 ^a^ |
| **TPs** |  |  |  |  | 1 |

ORAC: Oxygen radical absorbance capacity; H-ORAC: Hydrophilic oxygen radical absorbance capacity; L-ORAC: Lipophilic oxygen radical absorbance capacity; T-ORAC: Total oxygen radical absorbance capacity; TPs: Total phenolics; TE: Trolox equivalents. Values of IL-6 on continuous scales were log-transformed. ^a^ *P*<0.001 ^b^ 0.001≤*P*<0.01 ^c^ 0.01≤*P*<0.05.

**Supplementary Table 2. Comparison of the consumption of dietary H-ORAC contributing foods ^a^**

| **No.** | **Food (μmolTE/d)** | **Cumulative ^b^** | **Control (n=1312)** | **Case (n=654)** | ***P*-value ^c^** |
| --- | --- | --- | --- | --- | --- |
| 1 | Tea, green, brewed | 17.26 | 2656.69 ± 21251.16 | 342.36 ± 2133.84 | <0.001 |
| 2 | Grapes, black | 27.52 | 1548.26 ± 14015.52 | 263.59 ± 1611.35 | <0.001 |
| 3 | Orange juice, raw | 37.01 | 1192.58 ± 10727.20 | 725.42 ± 13503.65 | 0.44 |
| 4 | Apples, Fuji, raw, with skin | 45.43 | 1191.43 ± 2688.32 | 375.33 ± 713.10 | <0.001 |
| 5 | Apples, raw, with skin | 52.07 | 939.22 ± 2107.34 | 297.36 ± 561.49 | <0.001 |
| 6 | Strawberries, raw | 57.36 | 701.23 ± 1757.11 | 332.04 ± 640.60 | <0.001 |
| 7 | Sweet potato, raw | 62.16 | 747.34 ± 11533.99 | 76.11 ± 276.59 | 0.035 |
| 8 | Apples, Red Delicious, raw, with skin | 66.25 | 577.82 ± 1281.82 | 184.81 ± 344.70 | <0.001 |
| 9 | Oranges, raw, all commercial varieties | 70.23 | 500.63 ± 4358.52 | 304.26 ± 5530.46 | 0.43 |
| 10 | Radishes, raw | 73.33 | 378.78 ± 383.31 | 258.47 ± 195.30 | <0.001 |
| 11 | Potatoes, white, flesh and skin, raw | 75.93 | 295.45 ± 274.51 | 263.07 ± 169.34 | 0.0013 |
| 12 | Orange juice, canned, unsweetened | 78.46 | 317.71 ± 2717.35 | 193.02 ± 3461.11 | 0.42 |
| 13 | Plums, raw | 80.84 | 353.40 ± 1892.00 | 72.52 ± 273.62 | <0.001 |
| 14 | Bananas, raw | 82.93 | 301.90 ± 999.93 | 79.83 ± 226.07 | <0.001 |
| 15 | Peaches, raw | 84.55 | 241.10 ± 1270.56 | 50..60 ± 186.70 | <0.001 |
| 16 | Soybeans, mature seeds, sprouted, raw | 85.96 | 186.70 ± 372.82 | 87.34 ± 119.36 | <0.001 |
| 17 | Onions, raw | 87.23 | 137.00 ± 91.53 | 143.96 ± 67.10 | 0.06 |
| 18 | Tomato juice, canned | 88.50 | 177.98 ± 537.00 | 57.50 ± 118.70 | <0.001 |
| 19 | Lettuce, green leaf, raw | 89.71 | 139.91 ± 238.49 | 117.71 ± 126.57 | 0.007 |
| 20 | Lemon juice, raw | 90.61 | 142.38 ± 1784.21 | 10.82 ± 49.43 | 0.008 |

^a^ Adjusted for total energy intake using the residual methods. ^b^ Food items contributing to dietary H-ORAC intake that represented up to 90% of the cumulative contribution were selected. ^c^ *P*-values were calculated using Student’s *t*-test.

**Supplementary Table 3. Comparison of the consumption of dietary L-ORAC contributing foods ^a^**

| **No.** | **Food (μmolTE/d)** | **Cumulative ^b^** | **Control (n=1312)** | **Case (n=654)** | ***P*-value ^c^** |
| --- | --- | --- | --- | --- | --- |
| 1 | Spices, pepper, red or cayenne | 33.51 | 91.81 ± 107.09 | 36.79 ± 51.77 | <0.001 |
| 2 | Bananas, raw | 41.19 | 23.64 ± 72.10 | 4.82 ± 18.93 | <0.001 |
| 3 | Sweet potato, raw | 48.61 | 24.55 ± 323.57 | 2.35 ± 10.81 | 0.018 |
| 4 | Spices, pepper, black | 55.69 | 19.04 ± 24.74 | 8.25 ± 11.49 | <0.001 |
| 5 | Potatoes, white, flesh and skin, raw | 61.53 | 14.29 ± 13.26 | 8.79 ± 8.19 | 0.002 |
| 6 | Lettuce, green leaf, raw | 67.11 | 13.79 ± 22.82 | 8.21 ± 12.64 | 0.020 |
| 7 | Apples, raw, with skin | 70.04 | 8.81 ± 17.85 | 2.11 ± 5.22 | <0.001 |
| 8 | Apples, Fuji, raw, with skin | 72.88 | 8.55 ± 17.31 | 2.05 ± 5.07 | <0.001 |
| 9 | Peanuts, all types, raw | 75.70 | 8.81 ± 25.63 | 2.37 ± 6.63 | <0.001 |
| 10 | Galic, raw | 78.49 | 7.16 ± 4.65 | 3.71 ± 2.87 | <0.001 |
| 11 | Radishes, raw | 80.65 | 5.71 ± 5.77 | 2.68 ± 2.95 | <0.001 |
| 12 | Mushroom, oyster, raw | 82.69 | 5.56 ± 14.94 | 2.26 ± 6.00 | <0.001 |
| 13 | Watermelon, raw | 84.64 | 5.09 ± 11.33 | 2.51 ± 6.55 | <0.001 |
| 14 | Strawberries, raw | 86.39 | 4.87 ± 10.62 | 1.82 ± 4.32 | <0.001 |
| 15 | Apples, Red Delicious, raw, with skin | 88.04 | 4.94 ± 9.91 | 1.74 ± 2.93 | <0.001 |
| 16 | Peaches, raw | 89.42 | 4.32 ± 19.14 | 1.15 ± 3.38 | <0.001 |
| 17 | Carrots, raw | 90.70 | 2.48 ± 4.52 | 4.08 ± 8.01 | <0.001 |

^a^ Adjusted for total energy intake using the residual methods. ^b^ Food items contributing to dietary TPs intake that represented up to 90% of the cumulative contribution were selected. ^c^ *P*-values were calculated using Student’s *t*-test.

**Supplementary Table 4. Comparison of the consumption of dietary T-ORAC contributing foods ^a^**

| **No.** | **Food (μmolTE/d)** | **Cumulative ^b^** | **Control (n=1312)** | **Case (n=654)** | ***P*-value ^c^** |
| --- | --- | --- | --- | --- | --- |
| 1 | Tea, green, brewed | 16.85 | 2656.69 ± 21251.16 | 342.36 ± 2133.84 | <0.001 |
| 2 | Grapes, black | 26.85 | 1548.26 ± 14015.52 | 263.59 ± 1611.35 | 0.001 |
| 3 | Orange juice, raw | 36.11 | 1192.58 ± 10727.20 | 725.42 ± 13503.65 | 0.44 |
| 4 | Apples, Fuji, raw, with skin | 44.39 | 1201.49 ± 2711.56 | 378.43 ± 719.15 | <0.001 |
| 5 | Apples, raw, with skin | 50.95 | 949.76 ± 2131.56 | 300.62 ± 567.82 | <0.001 |
| 6 | Strawberries, raw | 56.16 | 707.46 ± 1773.17 | 334.90 ± 646.33 | <0.001 |
| 7 | Sweet potato, raw | 61.11 | 791.89 ± 12250.16 | 80.21 ± 292.18 | 0.036 |
| 8 | Apples, Red Delicious, raw, with skin | 65.14 | 583.56 ± 1294.84 | 186.61 ± 348.13 | <0.001 |
| 9 | Oranges, raw, all commercial varieties | 69.03 | 500.63 ± 4358.52 | 304.26 ± 5530.46 | 0.43 |
| 10 | Radishes, raw | 72.10 | 384.49 ± 389.09 | 262.36 ± 198.24 | <0.001 |
| 11 | Potatoes, white, flesh and skin, raw | 74.76 | 309.50 ± 287.57 | 275.56 ± 177.39 | 0.0013 |
| 12 | Orange juice, canned, unsweetened | 77.23 | 317.71 ± 2717.35 | 193.02 ± 3461.11 | 0.42 |
| 13 | Plums, raw | 79.56 | 354.51 ± 1898.16 | 72.73 ± 274.48 | <0.001 |
| 14 | Bananas, raw | 81.78 | 330.53 ± 1098.16 | 87.04 ± 246.88 | <0.001 |
| 15 | Peaches, raw | 83.42 | 248.31 ± 1310.15 | 52.03 ± 192.27 | <0.001 |
| 16 | Soybeans, mature seeds, sprouted, raw | 84.79 | 186.70 ± 372.82 | 87.34 ± 119.36 | <0.001 |
| 17 | Lettuce, green leaf, raw | 86.09 | 154.28 ± 263.31 | 129.64 ± 139.49 | 0.007 |
| 18 | Onions, raw | 87.35 | 138.98 ± 92.85 | 146.04 ± 68.07 | 0.06 |
| 19 | Spices, pepper, red or cayenne | 88.59 | 160.75 ± 188.36 | 93.26 ± 90.41 | <0.001 |
| 20 | Tomato juice, canned | 89.82 | 177.98 ± 537.00 | 57.50 ± 118.70 | <0.001 |
| 21 | Lemon juice, raw | 90.70 | 142.38 ± 1784.21 | 10.82 ± 49.43 | 0.008 |

^a^ Adjusted for total energy intake using the residual methods. ^b^ Food items contributing to dietary T-ORAC intake that represented up to 90% of the cumulative contribution were selected. ^c^ *P*-values were calculated using Student’s *t*-test.

**Supplementary Table 5. Comparison of the consumption of dietary TPs contributing foods ^a^**

| **No.** | **Food (mgGAE/d)** | **Cumulative ^b^** | **Control (n=1312)** | **Case (n=654)** | ***P*-value ^c^** |
| --- | --- | --- | --- | --- | --- |
| 1 | Tomato juice, canned | 13.88 | 116.80 ± 350.02 | 38.16 ± 78.53 | <0.001 |
| 2 | Apples, Fuji, raw, with skin | 24.70 | 90.80 ± 193.19 | 30.18 ± 53.87 | <0.001 |
| 3 | Apples, raw, with skin | 33.35 | 72.61 ± 153.75 | 24.24 ± 43.06 | <0.001 |
| 4 | Potatoes, white, flesh and skin, raw | 40.38 | 47.60 ± 44.19 | 42.48 ± 27.27 | 0.002 |
| 5 | Bananas, raw | 47.19 | 58.34 ± 183.15 | 16.62 ± 45.65 | <0.001 |
| 6 | Strawberries, raw | 53.47 | 48.97 ± 113.61 | 24.96 ± 43.92 | <0.001 |
| 7 | Apples, Red Delicious, raw, with skin | 58.75 | 44.20 ± 92.59 | 14.91 ± 26.19 | <0.001 |
| 8 | Sweet potato, raw | 63.59 | 44.50 ± 604.68 | 5.87 ± 19.03 | 0.021 |
| 9 | Cabbage, raw | 68.36 | 43.34 ± 166.99 | 6.69 ± 17.78 | <0.001 |
| 10 | Orange juice, canned, unsweetened | 70.99 | 19.74 ± 149.95 | 12.00 ± 194.22 | 0.37 |
| 11 | Radishes, raw | 73.37 | 17.34 ± 17.53 | 11.85 ± 8.96 | <0.001 |
| 12 | Catsup | 75.66 | 19.12 ± 55.64 | 6.55 ± 13.35 | <0.001 |
| 13 | Watermelon, raw | 77.91 | 16.34 ± 37.28 | 11.45 ± 21.15 | <0.001 |
| 14 | Raisins, seedless | 79.86 | 17.15 ± 134.68 | 3.90 ± 16.44 | <0.001 |
| 15 | Plums, raw | 81.46 | 13.90 ± 64.84 | 3.45 ± 10.82 | <0.001 |
| 16 | Peanuts, all types, raw | 82.96 | 12.96 ± 38.13 | 3.45 ± 9.84 | <0.001 |
| 17 | Peaches, raw | 84.45 | 13.00 ± 60.44 | 3.24 ± 10.13 | <0.001 |
| 18 | Lemon juice, raw | 85.85 | 13.01 ± 151.24 | 1.39 ± 6.02 | 0.006 |
| 19 | Lettuce, green leaf, raw | 87.13 | 8.75 ± 14.41 | 7.61 ± 8.04 | 0.025 |
| 20 | Spices, pepper, red or cayenne | 88.34 | 9.08 ± 10.41 | 5.37 ± 5.18 | <0.001 |
| 21 | Soybeans, mature seeds, sprouted, raw | 89.42 | 8.54 ± 16.05 | 4.21 ± 5.53 | <0.001 |
| 22 | Oranges, raw, all commercial varieties | 90.37 | 7.08 ± 51.18 | 4.31 ± 66.50 | 0.35 |

^a^ Adjusted for total energy intake using the residual methods. ^b^ Food items contributing to dietary TPs intake that represented up to 90% of the cumulative contribution were selected. ^c^ *P*-values were calculated using Student’s *t*-test.
