# Supplementary figures and images for: Circulating Interleukin-6 Level, Dietary Antioxidant Capacity, and Risk of Colorectal Cancer
Source: Antioxidants (Basel). 2019 Nov 28;8(12):595. doi: 10.3390/antiox8120595 (PMC6943549; doi:10.3390/antiox8120595)

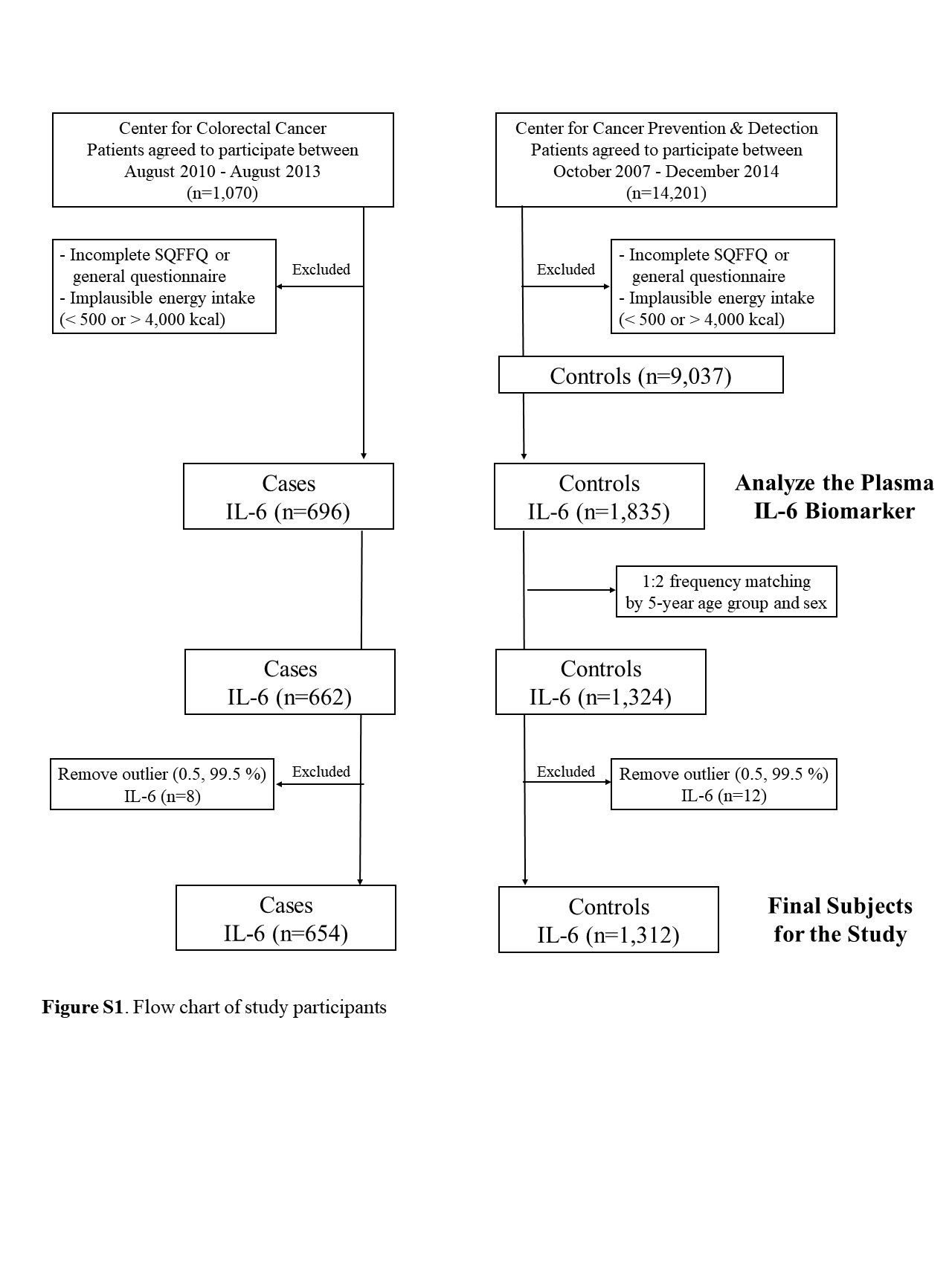

Supplement: Supplementary file 1 [file antioxidants-08-00595-s001.zip › 191127_Supplementary Figure S1_jmk.jpg]

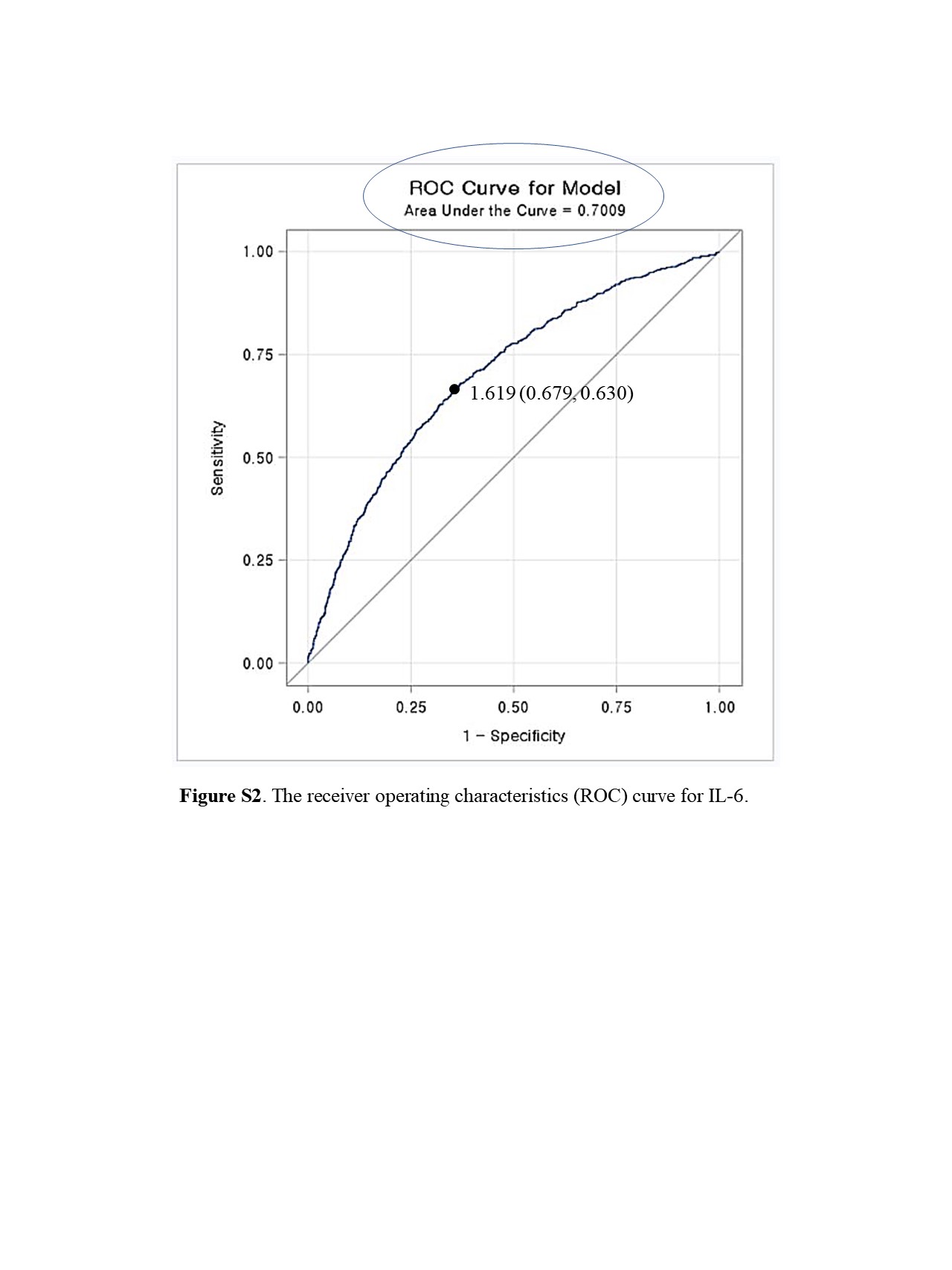

Supplement: Supplementary file 1 [file antioxidants-08-00595-s001.zip › 191127_Supplementary Figure S2_jmk.jpg]
